# Automated determination of hydrogen cyanide acrolein and total aldehydes in the gas phase of tobacco smoke

**DOI:** 10.1155/S1463924679000444

**Published:** 1979

**Authors:** W. S. Rickert, P. B. Stockwell

**Affiliations:** 1 Labstat Incorporated 262 Manitou Drive Ontario Kitchener N2C 1L3 Canada; 2 Laboratory of the Government Chemist Cornwall House Stamford Street London SE1 9NQ UK


### Automated determination of hydrogen cyanide acrolein and total aldehydes in the gas phase of tobacco smoke

**W.S. Rickert and P.B. Stockwell***

*Labstat Incorporated, 262 Manitou Drive, Kitchener, Ontario, Canada, N2C 1L3.*

For some time there has been wordwide concern for the health risks of cigarette smoking. In several countries, including the United States, Canada and the United Kingdom, surveys of the tar and nicotine delivered by commercial brands of cigarettes are carried out and published. Recently carbon monoxide levels have also been measured. The combustion of tobacco products such as cigarettes also produces three major ciliatoxic components, namely hydrogen cyanide (HCN), formaldehyde and acrolein [1]. As in the case of tar, nicotine and carbon monoxide, the detection of statistically significant differences requires the results of from 10 to 30 analyses per brand per chemical parameter at several points in time; as a consequence, the use of automated chemical analyses is advantageous. Individual automated colorimetric methods have been published for HCN, acrolein and total aldehydes using AutoAnalyser II techniques; however, the simultaneous determination of all three from a single sample has not been attempted due, in part, to the fact that different gas scrubbing agents and diluents must be used for each analyte. Selection of a single efficient scrubbing agent and the introduction of on-line dilution facilitate the simultaneous determination of the amount of HCN, acrolein and total aldehydes (mainly acetaldehyde) delivered by various brands of cigarettes. This improves the overall laboratory efficiency and minimises the manual effort in the analytical methodology.

### Equipment

In the present procedure the cigarette smoke is automatically generated using a Phipps and Bird 20-port smoking machine. After conditioning for 48 hours at 60% relative humidity and 22 °C each cigarette is lit and a puff of 35 ml is taken once per minute during 2 second intervals until a butt length of 30 mm is reached. Five cigarettes of a particular brand are smoked for each analysis.The particulate matter is trapped on a filter pad while the gas phase is passed through a sintered glass frit fitted into a trap behind the cigarette holder. The traps contain 35 ml of a suitable solvent (water, methanol or ethanol) thermostatted to 2 °C using a circulating bath containing 50% ethylene glycol and 50% water Immediately upon completion of the smoking 'run' of five cigarettes per sample, a 4 ml sample from each of the twenty traps is transferred into a sample cup which is sealed with a stretched piece of plastic film ensuring that volatile aldehydes are not lost whilst the samples are on the turntable awaiting analysis.The AutoAnalyser equipment used for the analyses consists of a dual probe sampler, a proportioning peristaltic pump (26 channel), three colorimeters, a three-pen recorder and a data processing system. The reactions employed are well established [2, 3, 4]: the determination of HCN is based on the Konnig reaction whereby cyanogen chloride (obtained from the reaction of chloramine T with HCN) reacts with pyridine to produce glutaconic aldehyde which in turn forms a coloured complex with pyrazolone; aldehydes are determined by reaction with 2, 4-dinitrophenylhydrazine to form substituted hydrazones which in turn produce coloured complexes with sodium hydroxide; acrolein is determined using the method developed by Cohen and Altshuller [5] whereby, in the presence of mercuric chloride and trichloracetic acid, acrolein condenses with 4-hexylresorcinol to give a blue complex. All three reactions are monitored colorimetrically.

* *Present address: Laboratory of the Government Chemist, Cornwall House, Stamford Street, London, SE1 9NQ, UK.*

**Table 1. Estimates of precision from the automated determination of hydrogen cyanide, acrolein and total aldehydes in standard aqueous solution and in methanolic extracts of the gas phase from a Canadian reference cigarette.**

| Standard | Concentration: nominal value (ppm) | Concentration: recorder response (mm) | Precision* (relative standard deviation,%) | Cigarette Delivery: mean (μg) | Cigarette Delivery: (relative standard deviation,%) |
|---|---|---|---|---|---|
| Acetaldehyde | 60.0 | 25 | 6.8 | | |
| | 120.0 | 51 | 8.1 | 861±67$^{\Delta}$ | 11 |
| | 180.0 | 76 | 8.8 | | |
| Hydrogen | 5.0 | 36 | 3.4 | | |
| Cyanide | 8.0 | 58 | 2.6 | 160±14 | 12 |
| | 10.0 | 72 | 3.3 | | |
| Acrolein | 5.0 | 37 | 6.1 | 58±4.6 | 11 |
| | 8.0 | 58 | 5.0 | | |
| | 10.0 | 73 | 2.9 | | |

* 120 observations
\+ 120 observations on 'Monitor D' over a period of 3 months.
Δ Confidence interval for 10 observations (P≥ .95)

**Table 2. The effect of trapping solution on the recovery of selected cigarette gas phase constituents.**

| Solvent$^{\Delta}$ | Constituent (μg/cigarette)*: HCN | ALDEHYDES | ACROLEIN |
|---|---|---|---|
| Water | 81 ± 5.5 | 718 ± 25 | 18 ± 1.5 |
| Methanol | 99 ± 7.1 | 830 ± 20 | 57 ± 4.6 |
| Ethanol | 95 ± 7.2 | 850 ± 39 | 52 ±3.8 |

* 10 observations on a Canadian reference cigarette were obtained.

ΔThe choice of solvents was based on the work of others who have used water [7] methanol [4] and ethanol [5] for extracting aldehydes, HCN, and acrolein respectively, in similar applications. Temperature was maintained at 2 ± 2 °C using a circulating bath and 50% ethylene glycol.

### Procedure

Figure 1 is a representation of the manifold used for simultaneously measuring the three analytes. The twin sampling probe incorporated in the sampler is used to deliver the sample stream to the manifold. One probe delivers the sample for the acrolein analysis whilst the other supplies a two-way stream splitter from which a sample is drawn for determination of HCN and total aldehydes. On-line mixing and sub-sampling must be carried out effectively and reproducibly. Simple use of coloured solutions enables the efficiency of the mixing assembly, shown in Figure 2, to be checked visually. Since there is a tendency for air introduced on sample switching to become trapped in the sub-sampling well, bubbles must be removed prior to this point as illustrated in figure 2.. The balance of flow rates used for the

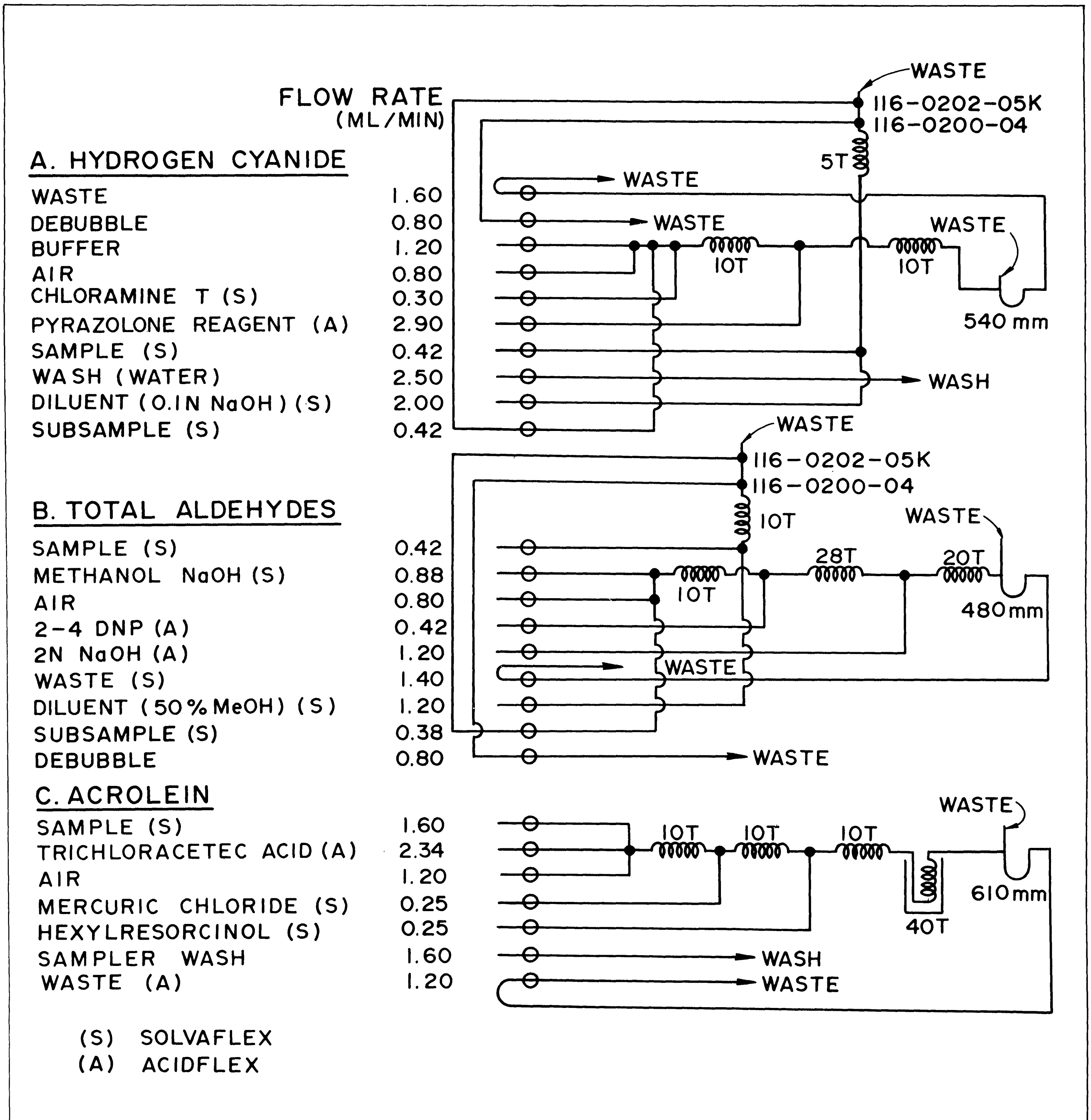

*Figure 1. Flow diagram of the analytical system used to determine the hydrogen cyanide acrolein and total aldehyde contents of a methanolic tobacco smoke extract.*

sample and dilution stream and the solubility and density of the stream are important parameters which must be matched for the reactions involved and the inclusion of a mixing coil just after diluent addition helps in this regard. For HCN the linear absorbance range of the colorimeter is 0 to 0.45 OD which corresponds to a level of 0 to 13 mg/kg. A similar range is also obtained for acrolein whilst the level for total aldehydes is 0 to 0.35 OD corresponding to 0 to 230 mg/kg. A suitable standard solution for each of these analytes is used to adjust the data processing equipment which provides a direct printout in concentration units. Once calibrated, the samples generated from the smoking machine can be analysed simultaneously for HCN, acrolein and total aldehydes with the results printed in the units $\mu g$ per cigarette. Although the individual methods are simple adaptations of the published procedures the integrated system as illustrated is unique in utilising a single sample with on-line dilution and sub-sampling. In this way, maximum responses are obtained and the anticipated levels of HCN and volatile aldehydes are reduced to within the range required for linear colorimetric response.

### Discussion

As already stated, the ability of the system to produce reliable determinations depends on efficient mixing and stable reaction chemistries. Table 1 is a summary of the performance of the system under realistic conditions over several

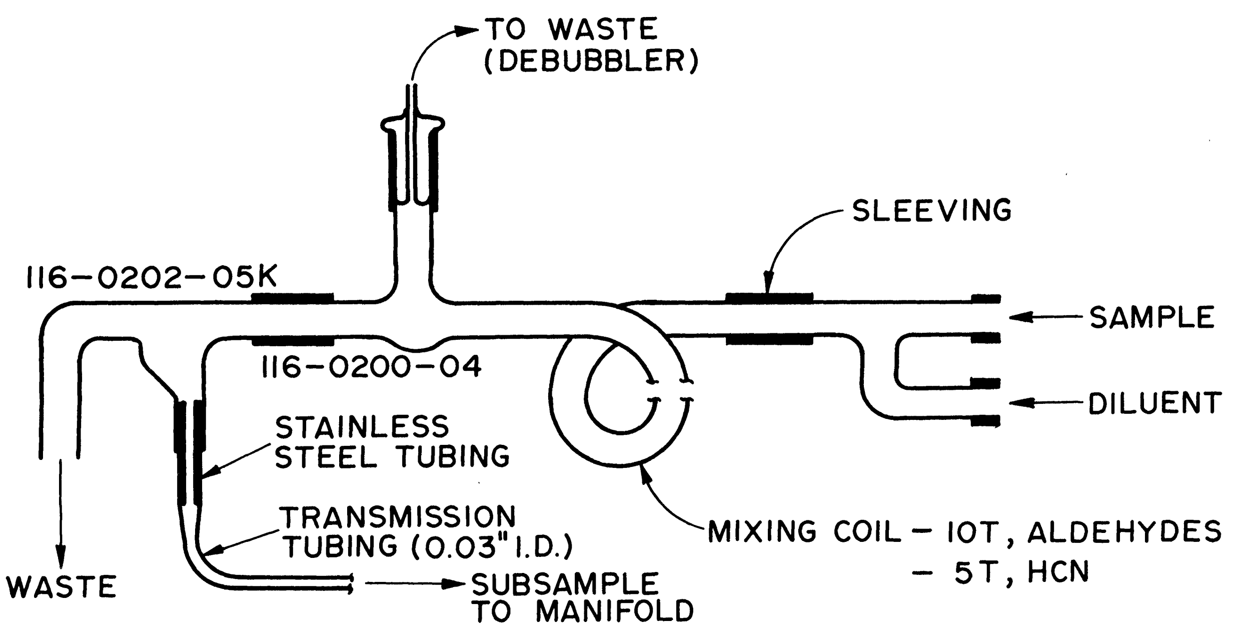

*Figure 2. The on-line dilution system.*

months. With respect to the standard solutions, at first glance the precision appears to be somewhat greater than the level of 2 - 4% reported by others for analyses of this type [6]. However, it must be remembered that estimates of variance include day-to-day variations in colorimeter response as well as week-to-week variations due to standard and reagent preparation. The variability in the yields from the 'monitor' cigarette are still larger due to the additional component of between-cigarette differences as a result of the manufacturing process. Accurate analytical results for the gas phase deliveries of cigarettes can be obtained only if the trapping solution efficiently absorbs the analytes under examination. The data in Table 2 indicates that either ethanol or methanol held at 2 °C can be used as a scrubbing agent for the extraction of HCN, acrolein and other volatile aldehydes. Experiments using a second flask connected in series demonstrated that the level of carry-over was slight. The maximum transfer was 4% obtained for total aldehydes using a water solvent; the minimum level of 2.6% was obtained for acrolein using ethanol as the solvent. It is therefore concluded that approximately 95% of the theoretical yield of the analytes are collected by a single trap.

### Conclusion

The automated analytical scheme briefly described represents a viable procedure for estimating the HCN, acrolein and total volatile aldehydes content in the gas phase of cigarette smoke and has improved the sample throughput in this laboratory. No special components are required. The approach is limited in its applicability to other methodologies only by the ingenuity of the systems designers the flexibility of the reactions involved and constraints due to the requirement of a multichannel system.

ACKNOWLEDGEMENT
This study was supported financially by Health and Welfare, Canada.

REFERENCES

[1] Battista, S.P. in 'Proceedings 3rd World Conference Smoking and Health' Ed. Wynder, E.L., Hoffman, D., and Gori, G.B., 1975, US Department of Health Education and Welfare Publication No. (NIH) 76-1221, p 517.
[2] Collins, P.F., Sarji, N.M., and Williams, J.F., *Beitrage Zur Tabakforschung,* 1973, 7, 73.
[3] Collins, P.F., Sarji, N.M. and Williams, J.F., *Tobacco Science,* 1970, **14**,182.
[4] Rothwell, K., and Grant, C.A. in 'Research Paper No. 11' 1974 and 1977, obtainable from Tobacco Advisory Council, London, UK.
[5] Cohen, I.R., and Altshuller, A.P., *Analytical Chemistry,* 1961, **33**, 726.
[6] Grannis, G.F., and Caragher, T.E., in 'Critical Reviews in Clinical Laboratory Science' 1977, 7, 327.
[7] Evans, D.J., and Mayfield, R.J., *Analyst,*1975,**100**,540.

